# “Vanishing” glioblastoma: A case report and review of the literature

**DOI:** 10.1016/j.radcr.2024.04.040

**Published:** 2024-05-18

**Authors:** Allegra Romano, Sara De Giorgi, Andrea Romano, Giulia Moltoni, Anna Maria Ascolese, Antonella Stoppacciaro, Alessandro Bozzao

**Affiliations:** a Neuroradiology Unit, Sant'Andrea Hospital, Department of Neuroscience, Mental Health and Sense Organs (NESMOS), Sapienza University of Rome, Rome, Italy; b Department of Radiotherapy, Sant'Andrea Hospital, Rome, Italy; c Department of Clinical and Molecular Medicine, Sant'Andrea Hospital, Sapienza University of Rome, Rome, Italy

**Keywords:** Vanishing glioblastoma, Corticosteroids, MRI

## Abstract

Contrast enhancement resolution induced by corticosteroids is a phenomenon primarily associated with primary central nervous system lymphoma, while malignant brain gliomas usually maintain a consistent radiological appearance during systemic steroid treatment.

Although rare, a few primary and metastatic intracranial lesions have shown similar radiographic changes following corticosteroid therapy. In the case of glioblastomas, corticosteroid therapy is commonly used to alleviate pressure effects from peritumoral edema, but its impact on contrast enhancement is not well-established.

A few reported cases in the literature describe reduced contrast enhancement in glioblastomas after corticosteroid treatment.

We present a case of corticosteroid-induced regression on imaging of glioblastoma evaluated at our institutionwith the intention to explore the pathogenesis of this response and discuss the therapeutic and prognostic implications of this discovery.

## Introduction

Malignant gliomas are prevalent primary brain tumors in adults, with an annual incidence of approximately 2–3 new cases per 100,000 individuals. These tumors typically manifest in the white matter of the frontal, temporal, or parietal lobes, with a typical intense peripheral contrast enhancement, varying degrees of central necrosis, and adjacent vasogenic edema on neuroimaging [1], [2], [3].

Steroid therapy is often employed to alleviate the pressure effects arising from peritumoral edema in glioblastomas [4]. However, its effect on contrast enhancement is not well-established or clearly understood. At times, the mass lesion may display a notable decrease in size when examined through contrast-enhanced magnetic resonance (MR) imaging or computed tomography (CT) scans. This characteristic is most frequently associated with primary central nervous system lymphoma (PCNSL) [5].

Nevertheless, there have been 8 reported cases of glioblastoma showing reduced contrast enhancement after the administration of corticosteroid treatment [1,^2^,[6], [7], [8], [9], [10].

In this context, we present a case of pseudo-regression on imaging of glioblastoma after corticosteroid therapy

## Case presentation

A 59-year-old female patient presented to our attention with a 10-day history of headache, nausea, spatio-temporal disorientation, and psychomotor slowing. Neurological examination revealed left hemianopsia. Blood tests were within normal limits. The patient has a family history of Lynch Syndrome.

**Fig. 1:**
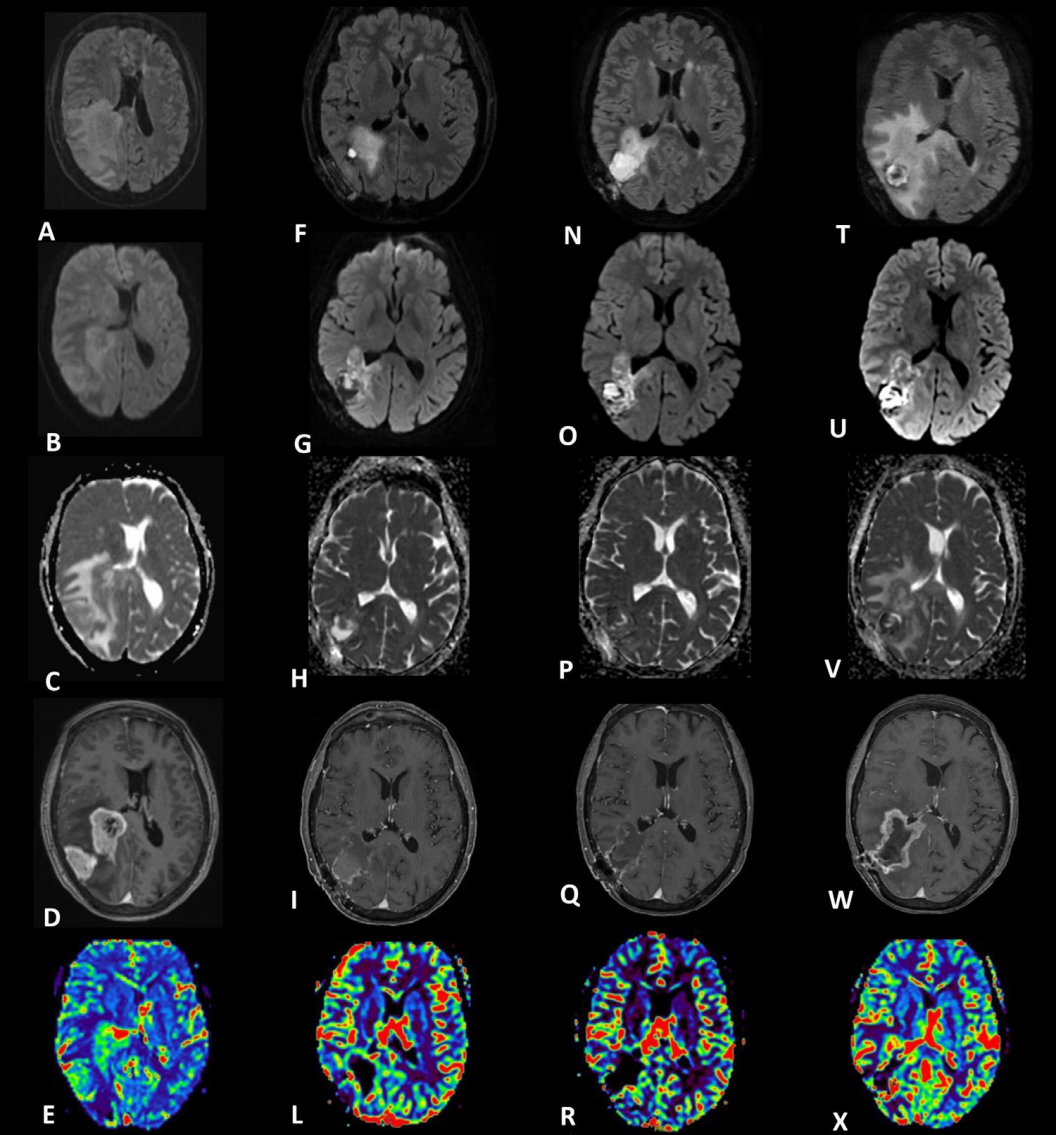
MRI investigations performed at our institution following tumor protocol comprising fluid attenuated inversion recovery (FLAIR) images (first row), diffusion weighted images (second row), apparent diffusion coefficient (third row), 3D fast spoiled gradient echo (FSPGR) post-contrastographic T1-weighted images (fourth row), dynamic susceptibility contrast perfusion images (fifth row). Initial magnetic resonance imaging on admission (A-E) 2 lesions in the right peritrigonal and temporo-parietal regions characterized by central necrotic areas, restricted diffusion (B and C), and post-contrastographic enhancement (D). Increased intra-lesional rCBV at DSC perfusion due to neoangiogenesis (E). Extensive perilesional edema with compressive effects on the right lateral ventricle and a 13 mm leftward midline shift (A). Brain MR scans after corticosteroid therapy (F-X). A follow-up scan at three weeks post biopsy and initiation of corticosteroid therapy (F-L) revealed a notable decrease in mass effect and contrast enhancement in the surgically untouched pathological tissue of the right peritrigonal region. Elevated perfusion parameters still present (L), with marked reduction of the vasogenic edema and consequently of the compressive effect on the ventricles, leading to a re-expansion of the ventricular trigone and a realignment of the midline (F). Follow-up MR 5 weeks after initiating corticosteroid treatment (N-R) showed a slight further decrease in the post-contrastographic enhancement at the periphery of the remaining pathological tissue (Q), with the other radiological findings remaining stable. Follow-up MR 9 weeks after tapering DEX down to its minimum dosage (2mg a day) and starting adjuvant radio-chemotherapy (T-X) showed tumor recurrence, evident by an increase in both size and extent of post-contrast enhancement (W). Increased perilesional edema (T) with a 5 mm left midline shift. Pathological increase in intralesional rCBV at DSC perfusion (X).

MR demonstrated the presence of 2 lesions of altered signal intensity with post-contrastographic enhancement localized in the right peritrigonal and temporo-parietal regions. These lesions showed central necrotic area, low apparent diffusion coefficient (ADC) values in solid components, and a pathological increase in perfusion parameters due to neoangiogenesis. Concomitant perilesional edema was present with compressive effects on the right lateral ventricle and a 13 mm leftward midline shift. MR findings were consistent with high-grade glial matrix lesions with multifocal localization (Figs. 1A-E).

**Fig. 2:**
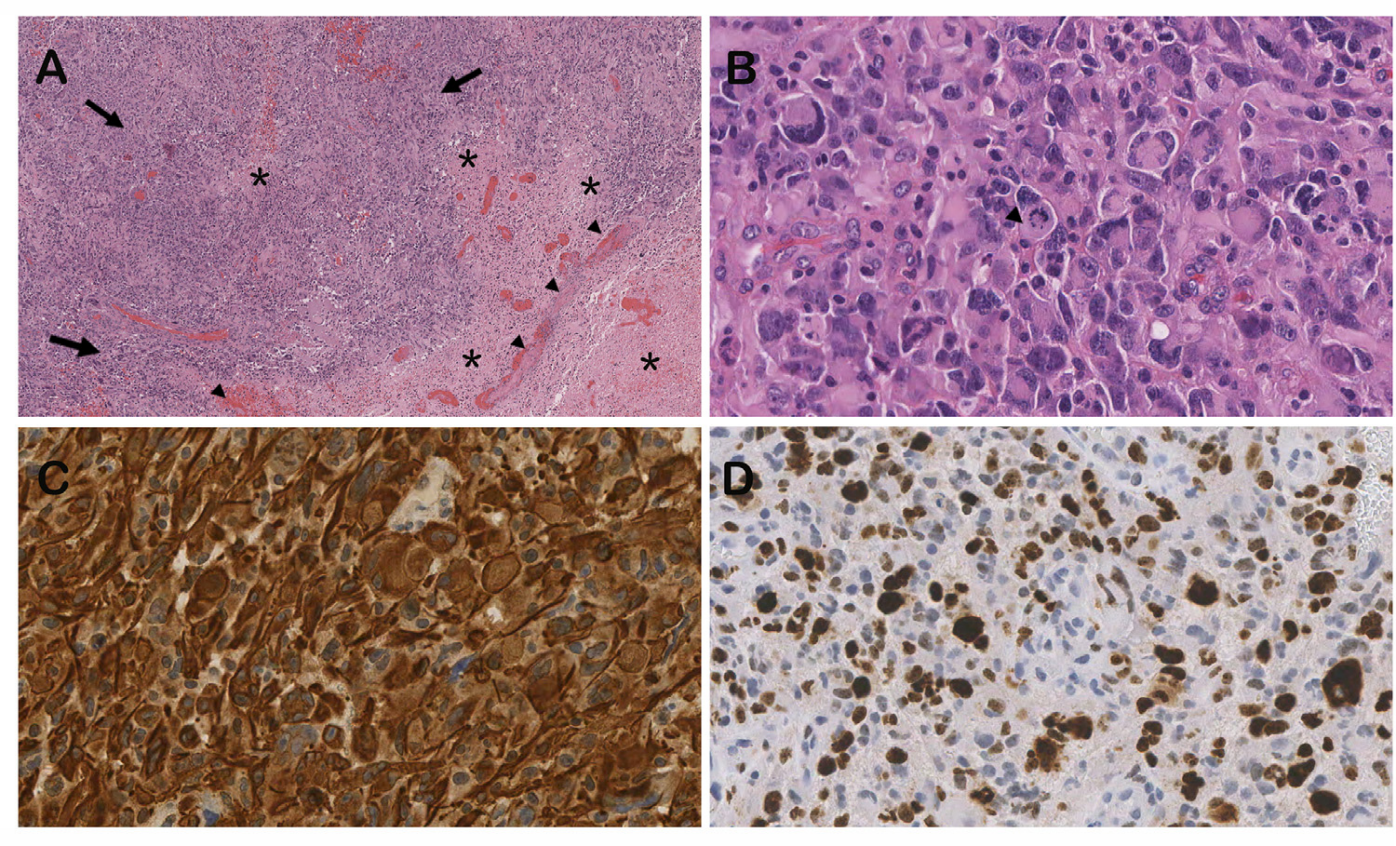
Glioblastoma characterization: at low power the tumor mass resulted constituted by dense cellular areas (arrow) mixed with prevalent hypoxic necrotic zones (asterisk) showing residual thrombotic vessels (Arrowhead) (A: H&E 100x enlargement). The tumor is histologically characterized by numerous multinucleated giant cells in a background of small astrocytes like cells. Atypical mitotic figures are frequent (arrowhead) (B: H&E 400x): GFAP immunostaining highlights the fusiform structure of the tumor cells (C: GFAP expression 400x). Ki67 immunostaining shows a high proliferative feature sustained by both giant and small cell component (D: Ki67 expression 400x).

A surgical procedure with excision of the superficial temporo-parietal lesion was performed. The histological analysis of the biopsy samples revealed dense cellular areas mixed with prevalent hypoxic necrotic zones, atypical mitotic figures, high proliferation at Ki67 immunostaining (Fig. 2), findings consistent with glioblastoma, immunophenotyped as IDH wild type, grade IV.

After the procedure, due to the residual perilesional edema and waiting for the start of radiotherapy, the patient was prescribed dexamethasone (DEX) (4 mg twice a day) and anti-epileptic agents.

A follow-up MR performed three weeks after surgery revealed a significant reduction in mass effect and contrast enhancement within the residual pathological tissue located in the right peritrigonal region, which appeared blurred and uneven. T2 hyperintensity and restricted diffusion persisted, along with increased perfusion parameters. The compressive effects were reduced with a re-expansion of the ventricular trigone and realignment of the midline (Figs. 1F-L).

The patient continued DEX and underwent a follow-up MR investigation 2 weeks later, indicating overall stability of the neuroradiological condition. A further modest reduction in the inhomogeneous peripheral post-contrastographic enhancement of the residual pathological tissue was observed, while the other radiological findings remained unchanged (Figs. 1N-R).

Then, corticosteroid treatment was reduced to its minimum dosage (2 mg/die) and adjuvant radio-chemotherapy began. A subsequent MR performed 9 weeks later disclosed tumor regrowth, evidenced by an expansion in both size and extent of post-contrastographic enhancement. Additionally, there was an increase in the perilesional edema with a 5 mm left midline shift and a pathological increase in perfusion parameters compared to the previous examination (Figs. 1T-X). Considering these findings, the overall picture suggested aggressive progression of the pathological glial tissue.

## Discussion

Glioblastomas typically do not show any changes in their radiological appearance or contrast enhancement pattern when subjected to steroid therapy [6].

Other pathologies exhibiting similar responses include lymphomas, inflammatory conditions, and tumor-like entities [2]. In particular, lymphomas are notably responsive to steroids, and corticosteroids alleviate neurological issues and decrease tumor size. The rapid elimination of neoplastic lymphocytes by steroids can quickly reduce lesion size or even their disappearance on contrast-enhanced scans within a few days. This rapid response may pose diagnostic challenges when a well-defined lymphoma mass is not visually identifiable [7]. Although extremely uncommon, also a few primary and metastatic intracranial lesions have been documented to display analogous radiographic changes following corticosteroid therapy [10].

In our case, the patient underwent a partial surgical excision of tumor before initiating any treatment, and the histological analysis confirmed the glioblastoma nature of the lesion (Glioblastoma IDH wild type, WHO grade IV).

Upon a thorough examination of the literature, we identified a limited number of cases describing such a response, commonly referred to as “vanishing glioblastoma” [1,^2^,[6], [7], [8], [9], [10] (Table 1).

**Table 1:** Contrast enhancement disappearance in glioblastomas: a summary of analogous cases reported in the literature.

Years	Author	Age/Sex	Location(s)	Multicentric	Radiographic change	Location of reappearance	Time to reappearance	Dexamethasone dose	Treatment	Clinical outcome	Molecular profile
**1997**	Buxton et al.	56M	Left frontoparietal	NO	Disappearance of lesion and enhancement	Same	3 weeks	6 mg per day, unspecified duration	None	Death immediately After biopsy	NR
**2004**	Zaki et al.	53M	Right parietal, splenium	YES	Reduced enhancement in parietal lesion, increased splenial enhancement	Same	3 weeks	16 mg per day for 3 weeks	Radiotherapy	NR	NR
**2004**	Zaki et al.	75M	Right parietal, splenium	YES	Reduced enhancement in parietal lesion, increased splenial enhancement	Splenium only	3 weeks	16 mg per day for 3 weeks	None	Death before radiation, unspecified timing	NR
**2009**	Hasegawa et al.	59M	Left parietal	NO	Reduced enhancement	Same	4 weeks	16 mg per day for 4 weeks	NR	NR	NR
**2009**	Goh et al.	61F	Right temporal, splenium	YES	Near resolution of all lesions	Same plus new right frontal lesion	4 weeks	16 mg per day for 4 weeks	Radiotherapy	Death 4 months after reappearance	NR
**2012**	Mazur et al.	57F	Right temporoparietal extending into splenium	NO	Reduced enhancement	Same plus leptomeningeal carcinomatosis	2 weeks	16 mg per day for 5 days	Radiotherapy, temozolomide	Alive 2 months after radiographic change	NR
**2012**	D'Elia et al.	66M	Right parietal extending into the splenium	NO	Reduced enhancement in the right parietal region	Same	10 days	8 mg/d	None	NR	NR
**2019**	Cuoco et al.	76F	Right parietal	NO	Reduced enhancement	N/A	N/A	16 mg per day for 5 weeks tapered	None	Death 1 month after surgery	Wildtype IDH1/2 Methylated MGMT Non-amplified EGFR Poor p53 expression
**2023**	Our case	59F	Right Temporoparietal and peritrigonal	YES	Reduced enhancement in the peritrigonal lesion	Same	7 weeks	8 mg per day for 9 weeks, then 2 mg/die (ongoing)	Radiotherapy, temozolomide	Alive after radiographic change	Wildtype GFAP+ ATRX+ IDH1- P53+ EGFR- Ki67 45%

n.r, not reported.

Steroid therapy is a well-established approach in the treatment of peritumoral edema associated with glioblastomas [11]. These agents can reduce blood-brain barrier permeability and achieve symptomatic improvement in a substantial percentage of brain tumor patients, ranging from 60% to 75%. DEX is the preferred steroid for treating peritumoral edema, chosen for its minimal salt retention and relative potency [4].

While not entirely understood, the exact mechanism through which brain post-contrast imaging is altered is thought to involve a decrease in blood-tumor barrier permeability, a reduction in tumor perfusion, diminished tumor diffusivity, and potentially an oncolytic effect on the tumor mass [10]. DEX plays a crucial role in maintaining BBB integrity by mitigating heightened permeability, restoring typical capillary states, and facilitating serum protein uptake into tumor cells [12]. It directly influences vasomotor states in pathological vessels and modulates nuclear glucocorticoid receptors, impacting tight junction proteins involved in BBB permeability [8]. Additionally, DEX reduces tumor perfusion by inhibiting Vascular Endothelial Growth Factor (VEGF) effects and tumor-produced VEGF, and it inhibits Granulocyte-Macrophage Colony-Stimulating Factor (GM-CSF) production [13]. It has been also hypothesized that DEX may have oncolytic effects acting on transcriptional modulation, leading to cellular damage and growth inhibition [12]. In our case, all previously reported effect of DEX were clearly evident, with a significant reduction of edema and a reduction of the blood-brain-barrier permeability, although the pathological condition of ADC and perfusion parameters suggested the persistency of tumor. DEX effects were similar to anti-angiogenetic therapy ones [14], leading to another example of pseudo-response of corticosteroid administration.

The mechanism driving this distinctive characteristic of anti-angiogenic therapy seems to be linked to the normalization of the blood-brain barrier (BBB) and a subsequent reduction in vascular permeability [15]. It is noteworthy, however, that there is no significant cytoreduction in tumor mass associated with this phenomenon [[16], [17], [18]. This unexpected result, reported in a few articles of literature, raised many questions about the practical usefulness of this finding and a reconsideration of this therapeutic approach [19,20], which is no longer regarded as the standard in clinical practice [14].

Considering these observed effects, our focus shifted to investigating whether this response could be dosage dependent. Existing literature on other cases of glioma disappearance on imaging post-corticosteroid therapy indicated a dosage range spanning from a minimum of 6 mg/d up to 16 mg/d, mostly utilizing a daily dose of 16 mg. DEX has been observed to down-regulate VEGF mRNA and protein expression in a dose-dependent manner both in normoxic and hypoxic conditions [[21], [22], [23].

Additionally, Wong et al. found that in the context of DEX usage in glioblastoma multiforme (GBM) patients, those administered higher doses of DEX (>4.1 mg daily) experienced notably shorter OS compared to their counterparts treated with lower doses (<4.1 mg daily) [4,24].

In our case, a follow-up evaluation was conducted to assess whether the maintenance of corticosteroid therapy could sustain its effects. The MR control 5 weeks after initiation of corticosteroids revealed modest modifications in the enhancement of the pathological tissue, suggesting a potential continued response to medical treatment. However, in the subsequent MR investigation, after tapering down corticosteroids, tumor regrowth was observed with findings suggestive of a high degree of aggressiveness. This outcome aligns with our expectations, considering that in the other reported cases, the tumor reappeared on imaging after 1–4 weeks after cessation of corticosteroid therapy and demonstrated increased aggressiveness in subsequent manifestations [1,^6^,7].

The use of DEX before chemoradiotherapy in gliomas is debated. Some authors advise against the use of steroids before chemotherapy. This recommendation is based on the observation that the reduced proliferation rate induced by cortisone therapy seems to provide a protective effect on cells, mitigating the oncolytic impact of certain cytostatic designed to target rapidly proliferating cells. On the other hand, regarding tumor radiotherapy, it seems that DEX exerts a protective effect [12]. This is attributed to its substantial reduction of capillary permeability within the tumor, consequently diminishing the extent of edema and enhancement associated with radiotherapy [12]. Other research studies have however indicated that the concurrent administration of DEX with radiotherapy has been associated with a reduction in cell death among cancer cells. This suggests a potential role of DEX in promoting radioresistance [25].

Similar doubts emerged considering the usefulness of these results after DEX in a neurosurgical planning. The reduced edema and mass effect could lead to an improvement of tumor resection, but few cases reported in literature are able in responding to this question [10].

In the existing literature, molecular genetic studies have only been performed in 1 of the 8 reported patients exhibiting imaging changes after corticosteroid treatment [2] (Table 1). It would be intriguing to explore whether there exist more specific histological patterns that could indicate which subtypes of glioblastomas may exhibit an imaging pseudo-response to corticosteroid treatment, although the interpretation of such response remains uncertain.

Another interesting aspect to evaluate could be the degree of survival of patients who show this pseudo-response to treatment with corticosteroids and the effectiveness of radiotherapy treatment to verify the suspected radio-resistance effect of corticosteroids. Only 3 of the 8 patients described in the literature have undergone radiotherapy treatment and the suspension of corticosteroids treatment alone seems to lead to a greater state of aggressiveness of the lesion.

## Conclusions

Steroid therapy has become a well-established approach in the management of peritumoral edema associated with glioblastomas. Notably, there are only 8 reported cases in the literature describing the disappearance of contrast enhancement in glioblastoma following the administration of high-dose oral steroids. Despite this, uncertainties linger regarding the utility of such a response; it remains a highly significant yet doubtful occurrence. Does this rare response suggest a potential advantage in adhering to classic treatment guidelines, given the inherent cortisone sensitivity of these tumors? Could it prove beneficial in surgical practice by facilitating tumor removal through edema reduction? Unfortunately, definitive answers to these inquiries are currently unavailable. Considering the effects of corticosteroids on the MR images and the dramatic recovery of the disease upon its suspension in the absence of visible effects of radiotherapy, the hypothesis of radio-resistance of DEX would be confirmed, worthy of further validation.

## Patient consent

I hereby confirm that I have obtained written informed consent from the patients to publish their cases.
